# Association Between Parameters of Penile Doppler Ultrasound and Cardiovascular Risk in Patients with Erectile Dysfunction: A Single-Center Retrospective Study

**DOI:** 10.3390/jcm15072722

**Published:** 2026-04-03

**Authors:** Andrea Graziani, Andrea Delbarba, Matteo Nardin, Nicola Caretta, Pierfrancesco Palego, Giuseppe Grande, Andrea Di Nisio, Carlo Cappelli, Alberto Ferlin

**Affiliations:** 1 Department of Medicine, University of Padova, 35128 Padova, Italy; andrea.graziani.1@unipd.it (A.G.); alberto.ferlin@unipd.it (A.F.); 2 Unit of Andrology and Reproductive Medicine, Department of Systems Medicine, University Hospital of Padova, 35122 Padova, Italy; nicola.caretta@aopd.veneto.it (N.C.); pierfrancesco.palego@aopd.veneto.it (P.P.); 3 Department of Clinical and Experimental Sciences, Struttura Semplice Dipartimentale di Endocrinologia, University of Brescia, Azienda Socio-Sanitaria Territoriale Spedali Civili, 25121 Brescia, Italycarlo.cappelli@unibs.it (C.C.); 4 Department of Biomedical Sciences, Humanitas University, Via Rita Levi Montalcini 4, 20090 Pieve Emanuele, Italy; matteo.nardin.89@gmail.com; 5 Internal Medicine, Department of Medicine, Azienda Socio-Sanitaria Territoriale Spedali Civili, Piazzale Spedali Civili 1, 25123 Brescia, Italy; 6 Department of Psychology and Health Sciences, Pegaso University, 80143 Naples, Italy; andrea.dinisio@gmail.com; 7 Centro per la Diagnosi e Cura delle Neoplasie Endocrine e delle Malattie della Tiroide, University of Brescia, 25121 Brescia, Italy

**Keywords:** erectile dysfunction, dynamic penile Doppler ultrasound, cardiovascular risk, QRISK3

## Abstract

**Background**: Erectile dysfunction (ED) is increasingly recognized as an early manifestation of systemic vascular disease and might represent a window for cardiovascular risk assessment. Dynamic penile colour Doppler ultrasound (PCDU) provides quantitative arterial and venous parameters that could reflect subclinical vascular impairment. We investigated the association between PCDU parameters and estimated cardiovascular risk in men with ED. **Methods**: In this single-center retrospective observational study, 275 men undergoing PCDU for ED were evaluated. Clinical characteristics, biochemical data, and QRISK3 10-year cardiovascular risk scores were collected. Mean peak systolic velocity (PSV), end-diastolic velocity (EDV), and resistive index (RI) were analyzed. Correlation analyses, logistic regression using a QRISK3 ≥ 10% threshold, linear regression models, age-stratified analyses, and receiver operating characteristic (ROC) curve analyses were performed. **Results**: Patients with impaired PSV (<35 cm/s) were older and exhibited higher QRISK3 scores and a greater prevalence of diabetes mellitus and previous cardiovascular events. Mean PSV was inversely correlated with QRISK3 (r = −0.203, *p* < 0.01) and was associated with higher cardiovascular risk categories in unadjusted logistic regression (β = −0.016, *p* = 0.048), but not after adjustment for age and diabetes mellitus. ROC analysis showed modest discrimination of increased cardiovascular risk (AUC = 0.60). The addition of PSV to a model including age and diabetes resulted in minimal improvement in discrimination (AUC 0.966 vs. 0.968). Age-stratified analysis demonstrated a significant association between lower PSV and higher cardiovascular risk only in patients ≤60 years. A progressive increase in QRISK3 was observed according to the number of abnormal Doppler parameters (*p* = 0.013). **Conclusions**: PCDU parameters reflect the overall cardiovascular risk burden in men with ED. Although not independent predictors beyond traditional risk factors, penile Doppler abnormalities might identify a vascular phenotype associated with higher estimated cardiovascular risk, particularly in younger individuals. These findings support the role of comprehensive vascular assessment in selected patients with ED.

## 1. Introduction

Erectile dysfunction (ED) is defined as the inability to achieve or maintain an adequate erection of the penis, to allow satisfactory sexual intercourse, for at least six months [1]. It is a common clinical condition, whose incidence and prevalence increase with age and is associated with worse general health and presence of other comorbidities. The incidence of ED is not truly known, which varies across studies, ranging from 4 to 66 cases per 1000 men/year [2]. The prevalence of ED varies based on the age of the enrolled subjects. According to the Massachusetts Male Ageing Study, ED has a prevalence of 52% in men aged 40–70 years [3], whilst the European Male Ageing Study showed a prevalence of ED ranging from 6 to 64%, with an average prevalence of 30% [4]. Besides the well-known—and easily understandable—sexual issues, ED is associated with classical non-communicable chronic diseases (NCDs), such as other andrological disorders such as male factor infertility. NCDs are usually defined as chronic diseases (i.e., cardiovascular diseases, respiratory diseases, diabetes mellitus…) which tend to be of long duration and are usually a result of genetic, physiological, environmental and behavioural factors. Indeed, the main reasons and risk factors for ED, such as smoking, physical inactivity, obesity, diabetes mellitus (DM), poor eating habits and disorders, abuse of alcohol and substances, are also main risk factors or causes of NCDs [5,6]—and it is interesting to underline that several of such risk factors are a risk factor for male factor infertility too [7,8]. Therefore, as suggested by Italian Guidelines dealing with ED, it is pivotal to approach patients with ED in a holistic approach, in light of a patient’s genomics, behaviour, risk factors and environment [2], as—indeed—it happens in any other fields of clinical practice. In particular, ED is common in patients with cardiovascular diseases (CVD) since the two conditions often share the same risk factors. Moreover, the presence of ED must be ruled out in patients with CVD and subjects with ED should be investigated to evaluate their cardiovascular risk, in the light of evidence that the appearance of ED allows for a time window (2–5 years) for earlier modification of associated risk factors [6]. The association between ED and CVD has been widely reported, with ED often preceding CV events and therefore being used as an early marker to identify men at high risk of major CVD, often in a subclinical condition [9]. Regarding the diagnosis of ED, besides accurate medical history, medical examination and biochemical evaluation, an important tool is represented by dynamic penile colour Doppler ultrasound (PCDU). Indeed, it is mostly used to evaluate the presence, the severity or the type of ED [10]. PCDU has also been reported to be useful in prediction of CVD. In fact, in a previous study from our group [11], it was reported that there was a strong association between cavernous arteries morphological alterations and CVD with a threefold increased risk of future CVD in comparison to patients with healthy cavernous arteries (RR 3.2, 95% CI 1.17–8.78).

PCDU is performed with the patient in the supine position. It requires the intracavernous injection (ICI) of alprostadil, which is synthetic prostaglandin E1 acting as vasodilating agent, further leading to erection. PCDU lets an accurate evaluation of the structures of the penis, the vessels, penile tissues and quantitative vascular parameters, in particular peak of systolic velocity (PSV) of cavernous arteries. The PSV indicates the maximum flow rate detectable in systole in the various samples performed during the examination [10]. Other vascular parameters evaluated are end-diastolic velocity (EDV), indicating the amount of flow still present in the artery at the end of the diastolic phase. and resistive index (RI), resulting from the PSV-EDV/PSV formula and expressing the degree of peripheral resistance to flow. A large (and growing) body of evidence has shown that impaired dynamic PSV after PGE1 stimulation might be considered a marker of systemic vascular damage and an early indicator of impending major adverse cardiovascular events (MACE). Interestingly, this association appears to be particularly strong in younger (40–60 years of age) patients with unrecognized cardiovascular risk factors, whereas in older individuals the predictive value of PCDU is attenuated by the presence of multiple comorbidities. Therefore, patients aged between 40 and 60 years presenting with ED might be considered for PCDU. This aspect has been confirmed in the latest Princeton Consensus Guidelines [12].

On the other hand, PCDU might present some risks associated with the injection of alprostadil, especially the risk of priapism, although this risk might be low and often preventable [13].

The aim of this study was to evaluate dynamic parameters of PCDU as a parameter associated with estimated cardiovascular risk burden and evaluating a possible correlation between biochemical parameters and cardiovascular risk of patients with ED and the said dynamic parameters.

## 2. Materials and Methods

This was a single-center retrospective observational study, evaluating patients referred for evaluation of suspected vascular ED undergoing PCDU in the Unit of Andrology and Reproductive Medicine, Department of Medicine, University of Padua, Italy, between January 2021 and February 2024.

For each patient, we collected data regarding medical history (personal and familial anamnesis, drugs, etc…), biochemical parameters (general, hormonal and metabolic) and QRISK3 score—10 years (calculated using a proper calculator) for patients from 25 to 84 years old without previous cardiovascular events (PCVE).

ED was diagnosed based on clinical history and andrological evaluation performed during the outpatient visit. Due to the retrospective nature of the study and the long study period, standardized questionnaires—validated measure for the assessment of ED severity (such as International Index of Erectile Function—IIEF-5, Erection Hardness Score—EHS, or other validated instruments)—were not systematically available for all patients and therefore were not included in the present analysis.

In detail, regarding patients’ history, we focused on data regarding cardiovascular risk factors, such as DM, arterial hypertension, hypercholesterolemia, obesity, hypogonadism, metabolic syndrome (MetS), hypothyroidism, smoking habit and PCVE (considered as major adverse cardiovascular events). Cardiovascular risk factors were defined based on documented clinical diagnosis (for DM: according to glucose levels or HbA1c levels; for arterial hypertension: according to overall cardiovascular risk factors; for dyslipidemia: according to overall cardiovascular risk factors and cLDL concentrations; for obesity: according to BMI levels; for MetS: according to IDF criteria) or ongoing pharmacological treatment recorded in the medical charts. DM, arterial hypertension, dyslipidemia, obesity, MetS, smoking status and PCVE were recorded as part of the routine clinical evaluation. Additional comorbidities and medication history were retrieved when available in clinical records.

As stated, patients were referred for evaluation of suspected vascular ED in a tertiary andrology setting. Due to the retrospective design, a standardized etiological classification was not systematically available. However, patients with a clear non-vascular etiology of ED (including overt psychogenic ED, situational ED, major psychiatric disorders, neurological diseases, or recent prostate surgery) were not routinely referred for PCDU in our clinical practice and were therefore unlikely to be included in the study cohort. Consequently, the present population can be considered enriched for patients with suspected vascular ED, although a formal etiological classification could not be systematically applied.

### 2.1. QRISK3 Score and Biochemical Evaluations

We calculated the overall prevalence of patients with PCVE in our cohort of patients, further excluding such patients (with previous PCVE) from the evaluation of QRISK3 score. Regarding biochemical evaluation, we considered a concentration of total testosterone (TT) below 12 nmol/L and/or a concentration of calculated free testosterone (cFT)—according to Vermeulen formula—below 0.220 nmol/L as suggestive for hypogonadism, according to most recent guidelines [14]. Luteinizing hormone (LH) and follicle-stimulating hormone (FSH) were considered to be normal when included in values between 1 and 9.4 and 1–8, respectively. Thyroid-stimulating hormone (TSH), evaluated with chemiluminescence, was considered to be normal when included between 0.4 and 4 mIU/L. Hormonal evaluations were performed at morning, after overnight fasting, between 8 am and 10 am [15].

### 2.2. Penile Colour Doppler Ultrasound (PCDU)

PCDU was executed by two experienced ultrasound specialists working in our Unit (NC and PF), following a standardized protocol, in a similar and standardized way, as previously described [10]. The examinations were performed following a standardized procedure including intracavernous alprostadil injection, sequential Doppler measurements of PSV, EDV, and RI, and consistent timing of measurements during the erectile response. It was performed using intracavernous injection of PGE1 (alprostadil) at an average dosage of 10 mcg, according to the clinical characteristics of the patients [13]. In patients considered at higher risk of priapism (e.g., younger patients, neurological disorders, or recent prostate surgery), an initial dose of 5 µg was administered, with the possibility of redosing to reach a total dose of approximately 10 µg if the erectile response was insufficient. In routine clinical practice, the administered dose could vary depending on patient characteristics, generally ranging between 2 and 20 µg.

We collected data regarding the percentage of erective response to alprostadil (expressed in %), and data regarding PSV (expressed in cm/s as a mean of PSV obtained measuring at the origin of both right and left cavernous arteries). For the percentage of erective response to alprostadil, we divided the response in four categories: 1 (0–10%), 2 (11–50%), 3 (51–75%) and 4 (76–100%). For mean PSV, we considered a value above 35 cm/s as normal and a value below 35 cm/s as pathological, without further sub-classifications in patients with pathological PSV. This threshold is commonly used in clinical practice to identify arterial insufficiency during PCDU and corresponds to the cut-off routinely adopted in our clinical unit. Regarding other vascular parameters, we collected data regarding EDV (expressed in cm/s, normal value below 3 cm/s) and RI (expressed as a number, normal value above 0.9). Negative EDV values were considered indicative of adequate veno-occlusive function.

The study size was determined by the number of patients referred to for PCDU during the study period who met the inclusion criteria. No formal sample size calculation was performed because of the retrospective design.

The study protocol follows the standard clinical approach and the principles outlined in the Declaration of Helsinki. Informed consent to collect the data anonymously for the scientific purpose was obtained from the study participants.

### 2.3. Statistics

Statistical Package for the Social Sciences software IBM SPSS Statistics, Version 29.0, (IBM, Armonk, NY, USA) was used for statistical analysis. The entire cohort was grouped according to the mean PSV value, <35 cm/s and ≥35 cm/s. Continuous variables are expressed as median and interquartile (IQR) range, while categorical as percentage. For continuous variables, Mann–Whitney U test was used for comparison between two groups, while Kruskal–Wallis test was used when more than two groups were compared. Differences between categorical variables were analyzed with the Chi-square test, or with Fisher’s exact test as appropriate. Correlations were obtained using Spearman’s coefficient between QRISK 10, PSV and response to PGE1 and main PCDU parameters and laboratory values. In addition, cardiovascular risk was categorized according to QRISK3 thresholds, defining patients with QRISK3 ≥ 10% as having increased cardiovascular risk. Binary logistic regression analysis was performed to evaluate the association between dynamic PCDU parameters (mean PSV, EDV and RI) and the presence of increased cardiovascular risk. Linear regression analysis was used to inquire the role of main PCDU parameters variations to predict QRISK3 changes. A test for trend was performed inquiring the relation between the number of impaired PCDU parameters and QRISK3. To further explore the clinical discriminatory ability of penile Doppler parameters, receiver operating characteristic (ROC) curve analysis was performed, and the area under the curve (AUC) was calculated to assess the performance of mean PSV in identifying patients with higher cardiovascular risk (QRISK3 ≥ 10%). Continuous Doppler parameters were analyzed using mean values obtained from both cavernous arteries. An age-stratified analysis was performed by dividing patients into ≤60 and >60 years groups, in order to explore potential effect modification by age. To formally assess effect modification by age, an interaction term between age and mean PSV was included in the multivariable logistic regression model. Logistic regression models were separately fitted in each subgroup. Age and diabetes mellitus were included in multivariable models because they represent major determinants of cardiovascular risk and potential confounders of penile vascular parameters. To evaluate the incremental predictive value of mean PSV beyond traditional cardiovascular risk factors, ROC curves were generated for a base model including age and diabetes mellitus and for an extended model additionally including mean PSV. The AUC values were compared descriptively. Collinearity between variables included in multivariate models was assessed by variance inflation factor (VIF). The primary outcome of the study was increased cardiovascular risk defined as QRISK3 ≥ 10%. The main exposure variables were PCDU parameters (mean PSV, EDV and RI). Age and diabetes mellitus were considered the main confounding variables and were included in multivariable analyses. The objective of these analyses was not to develop a predictive model for QRISK3 but rather to explore the association between penile vascular parameters and estimated cardiovascular risk categories. A *p* value < 0.05 was considered statistically significant.

## 3. Results

A total of 275 patients underwent penile Doppler ultrasound during the study period. QRISK3 score was available for 253 subjects, who were included in the main analyses (22 excluded due to age outside the eligible range or presence of previous cardiovascular events). Among them, 158 patients were aged ≤60 years and 95 were aged >60 years. A total of 270 patients had complete Doppler parameters available (five were excluded due to missing data). The flow-diagram describing patients’ inclusion and data availability according to STROBE recommendations is summarized in Supplementary Figure S1.

Table 1 describes general data regarding the patients enrolled and the differences in such parameters according to the PSV value (below or above the cut-off of 35 cm/s) (Table 1).

In particular, we reported that patients with normal PSV values (above 35 cm/s) were younger (55 (48–64) vs. 62 (54–67) years, *p* = 0.001), had a lower cardiovascular risk according to QRISK3 (10.0 (5.5–15.5) vs. 13.6 (10.1–14.6), *p* = 0.006) and a lower prevalence of PCVE (4.2% vs. 13.6%, *p* = 0.008) and DM (7.4% vs. 16.9%, *p* = 0.026), had a higher RI (0.95 (0.86–1.00) vs. 0.82 (0.77–0.95), *p* < 0.001) and response to PGE1 (83 (70–100) vs. 65 (50–80), *p* < 0.001). We reported no differences when dealing with prevalence of other cardiovascular risk (hypertension, dyslipidaemia, obesity, MetS), nor in the prevalence of hypogonadism, nor in the prevalence of parameters such as BMI and metabolic biochemical profile. Although total testosterone levels were comparable between the two groups, calculated free testosterone levels were significantly lower in the group with pathological PSV (0.20 (0.13–0.26) vs. 0.24 (0.19–0.31) nmol/L, *p* = 0.040).

Only few anthropometric parameters were available for a subset of patients (BMI and blood pressure measurements). In detail, median BMI was 28.7 kg/m^2^ (IQR 25.6–32.2), systolic blood pressure was 140 mmHg (IQR 125–145) whilst diastolic was 85 mmHg (80–90).

In Table 2 we reported the correlations between the studies variables and QRISK3, mean PSV and response to PGE1.

Focusing on PCDU parameters, we found significant correlations when evaluating the age of patients (positive correlation with QRISK3, negative correlation with mean PSV and response to PGE1, *p* < 0.01), QRISK3 (negative correlation with mean PSV and response to PGE1, *p* < 0.01), mean PSV (negative correlation with QRISK3, positive correlation with response to PGE1, *p* < 0.01), response to PGE1 (negative correlation with QRISK3, positive correlation with mean PSV, *p* < 0.01) and other PCDU parameters. In particular, when evaluating EDV and RI, we found that they were both correlated (positive and negative correlation, respectively) with QRISK3 (*p* < 0.01) (Table 2).

**Figure 1:**
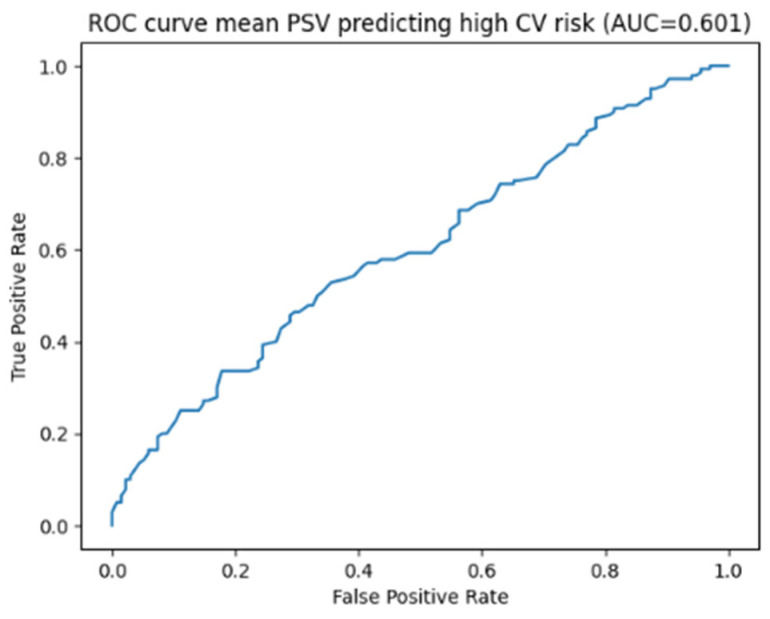
ROC analysis on mean PSV predicting high cardiovascular risk.

When cardiovascular risk was categorized using a QRISK3 threshold of 10%, binary logistic regression analysis showed that lower mean PSV values were significantly associated with a higher cardiovascular risk category (β = −0.016, *p* = 0.048), whereas EDV and RI were not significantly associated with increased risk. Receiver operating characteristic (ROC) curve analysis demonstrated a modest ability of mean PSV to discriminate patients with increased cardiovascular risk, with an area under the curve (AUC) of 0.60 (95% CI 0.53–0.67) (sensitivity and specificity at the 35 cm/s threshold) (Figure 1). In particular, using the predefined threshold of 35 cm/s, sensitivity was 27% (95% CI 20.5–35.0%) and specificity was 89% (95% CI 82.4–93.8%). The high discriminative performance observed in the base model reflects the intrinsic structure of the QRISK3 score, which is strongly driven by variables such as age and diabetes mellitus.

However, after adjustment for age and presence of DM, the association between mean PSV and increased cardiovascular risk was no longer significant, suggesting that the observed association might be largely driven by baseline cardiovascular risk factors.

When stratifying patients according to age (≤60 years, n = 158–162.5%; >60 years, n = 95–137.5%), the association between mean PSV and increased cardiovascular risk (QRISK3 ≥ 10%) remained significant only in younger patients (β = −0.023, *p* = 0.041), whereas no significant association was observed in patients older than 60 years (*p* = 0.51). To formally evaluate effect modification by age, an interaction term between age and mean PSV was included in the multivariable logistic regression model. The interaction term was statistically significant (β = 0.005, *p* = 0.006), confirming that the association between PSV and increased cardiovascular risk varies according to age. In particular, the inverse association between PSV and cardiovascular risk appeared stronger in younger individuals and progressively attenuated with advancing age.

To assess the incremental value of mean PSV beyond traditional risk factors, we compared a base model including age and diabetes mellitus with a model additionally incorporating mean PSV. The addition of PSV resulted in a minimal increase in discriminative performance (AUC 0.966 vs. 0.968), indicating that while PSV reflects overall cardiovascular burden, its independent contribution to global risk prediction appears modest.

**Figure 2:**
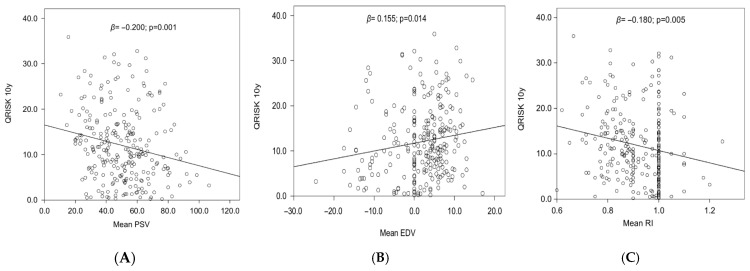
Regression analysis evaluating the association between PCDU parameters ((**A**): PSV, (**B**): EDV and (**C**): RI) and QRISK3.

Subsequently, we performed linear regression analysis evaluating the association between PCDU parameters and QRISK3. All three PCDU parameters evaluated significantly predicted QRISK3 values at univariate analysis (Figure 2A–C).

After correction for main baseline differences, the PCDU parameters were not confirmed independent predictors of QRISK3 (Supplementary Materials, Table S1).

**Figure 3:**
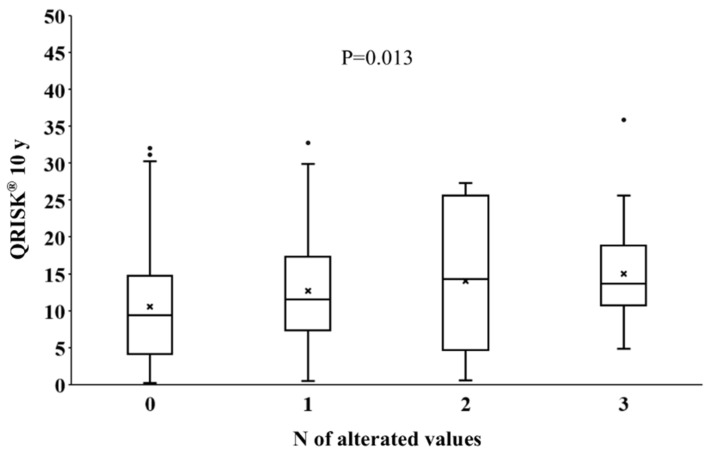
Association between QRISK3 and number of pathological PCDU parameters.

In our cohort, 270 patients (98.2%) displayed all three PCDU parameters available. We found 137/270 (50.74%) patients without any pathological PCDU value, 91/270 (33.70%) with one pathological value, 22/270 (8.15%) with two pathological values and 20/270 (7.41%) with three pathological values. To this point, we decided to perform a further analysis in order to evaluate the association between QRISK3 and the number of pathological PCDU parameters, PSV, EDV and RI: we found a significant higher QRISK3 as the number of pathological PCDU increased per patient (*p* = 0.013) (Figure 3).

## 4. Discussion

ED is a very common male sexual dysfunction, which might be caused by several risk factors, mostly shared with cardiovascular events. Associations between ED, MACE and altered PSV parameters appear to be particularly strong, as previously said, in younger patients with unrecognized cardiovascular risk factors, whilst in older individuals the predictive value of PCDU is attenuated by the presence of multiple comorbidities. Therefore, patients aged between 40 and 60 years with ED might be particularly suitable candidates for comprehensive vascular evaluation including PCDU.

Increasing evidence suggests that vasculogenic ED should be interpreted within the broader framework of systemic vascular and inflammatory dysfunction rather than as a purely localized penile disorder. Endothelial dysfunction, oxidative stress, and chronic low-grade inflammation might, in fact, play a central role in the pathophysiology of vascular ED, being also key mechanisms in atherosclerosis development. In this context, ED might represent an early clinical manifestation of systemic vascular impairment, reflecting the same pathophysiological processes underlying cardiovascular disease. This concept supports the interpretation of penile vascular alterations detected by Doppler ultrasound as markers of systemic vascular dysfunction rather than isolated local abnormalities [16,17].

In order to evaluate the association between ED and CVD risk, we calculated the 10-year cardiovascular risk using the QRISK3 score and evaluated the pathologies present in the medical history of patients, biochemical parameters (dealing with metabolic and hormonal profile) and the vascular parameters obtained by PCDU. We chose the QRISK score when dealing with CVD risk of our cohort of patients because it is an easy, simple and accurate cardiovascular risk score to estimate 10-year cardiovascular risk.

The threshold of 35 cm/s used in the present study deserves clarification. In fact, if several studies have proposed lower cut-offs (approximately 25–30 cm/s) to define severe arterial insufficiency in the context of ED (see also below), in clinical practice a threshold of 35 cm/s is frequently adopted as an indicator of impaired arterial inflow during PCDU. The choice of this value in our study reflects routine clinical practice in our tertiary andrology unit rather than an attempt to redefine the optimal diagnostic threshold. Therefore, our results should be interpreted within the framework of clinical vascular assessment rather than as a proposal of a new diagnostic cut-off. However, the present data should not be interpreted as evidence supporting the superiority of this cut-off compared to lower thresholds (e.g., 25 cm/s) reported in the literature, as no direct comparison was made and the overall discriminative performance was modest.

In our study, we found that patients with pathological PSV values (below 35 cm/s), in respect to patients with normal PSV values, were older, had higher cardiovascular risk according to QRISK3 score, higher RI, higher prevalence of PCVE and DM, lower response to PGE1 administration during PCDU and low cFT concentrations. On the other hand, we found no differences when evaluating the prevalence of well-studied cardiovascular risk factors (arterial hypertension, obesity, dyslipidaemia and MetS) nor in the values of BMI, glycaemia and lipidic profile. Although this finding might appear unexpected, several factors could contribute to this observation. First, the relatively limited sample size could have reduced the statistical power to detect moderate associations. Second, the study population consisted of patients referred to a tertiary andrology center for evaluation of ED, which might represent a selected population with a high prevalence of multiple overlapping risk factors. Finally, the strong influence of age on both penile vascular parameters and cardiovascular risk estimation could have overshadowed the contribution of other individual metabolic factors.

According to data obtained, we observed that PSV might represent a marker associated with the cardiovascular risk profile of patients with ED, since a negative correlation was reported with statistical significance, indicating that as PSV decreases the 10-year cardiovascular risk of the patient considered increases. Therefore, we suggest that when analyzing a patient with ED or suspicion of ED with PCDU it might contribute to the clinical estimation of cardiovascular risk profile. Furthermore, PSV appeared among PCDU parameters most strongly associated with QRISK3 as expected, given that the higher prevalence of cardiovascular pathologies with age, representing the major risk factor [18]. Although lower PSV values were associated with higher estimated cardiovascular risk in unadjusted analyses, this relationship was markedly attenuated after adjustment for age and diabetes mellitus. This finding suggests that the observed association between PSV and cardiovascular risk might partly reflect the physiological decline of penile arterial flow with advancing age, which parallels the age-driven increase in cardiovascular risk scores.

Notably, although lower PSV values were associated with higher cardiovascular risk categories in unadjusted analyses, this association was attenuated after adjustment for age and DM, suggesting that PCDU parameters might primarily reflect the underlying cardiovascular risk burden rather than acting as independent determinants. Since QRISK3 incorporates several cardiovascular risk factors (including blood pressure, lipid profile, and diabetes), additional adjustment for all these variables would have resulted in substantial collinearity and potential over-adjustment. For this reason, multivariable models were intentionally limited to age and diabetes mellitus, which represent the most clinically relevant determinants of cardiovascular risk and potential confounders of the association between penile vascular parameters and estimated cardiovascular risk. This approach allowed us to control for the strongest clinical confounders while avoiding excessive adjustment for variables already embedded in the risk score algorithm. When evaluating the incremental predictive value of mean PSV beyond age and diabetes mellitus, only a minimal improvement in model discrimination was observed. This finding suggests that penile Doppler parameters primarily reflect the overall cardiovascular risk burden rather than substantially enhancing traditional risk prediction models. However, their potential value might lie in the identification of a vascular phenotype in selected subgroups rather than in improving global risk score performance.

Finally, when stratifying patients according to age (≤60 vs. >60 years), the association between mean PSV and increased cardiovascular risk remained significant only in younger patients (β = −0.023, *p* = 0.041), whereas no significant association was observed in patients older than 60 years (*p* = 0.51). The formal interaction analysis further supports the hypothesis that penile arterial impairment might carry greater clinical relevance in younger individuals. These findings suggest that the relationship between penile arterial impairment and estimated cardiovascular risk is more pronounced in younger individuals, supporting the hypothesis that dynamic PCDU parameters might identify a subclinical vascular phenotype particularly relevant in earlier stages of cardiovascular disease. These findings reinforce the concept that penile Doppler abnormalities may serve as an early vascular marker particularly in younger men, whereas in older individuals the predictive signal is largely overshadowed by established risk factors.

These results are in line with several studies reported in the literature. In particular a negative correlation was found, as in our study, between PSV value and cardiovascular risk [19,20]. Indeed, patients with a normal ultrasound examination had an unlikely risk of coronary heart disease, whereas patients with an ultrasound with an abnormal response had obstructive coronary disease in 30% of cases [17].

However, there are factors to consider, in particular the fact that PCDU is an operator-dependent diagnostic test, as well as being expensive and time-consuming. For these reasons, it may not be an optimal parameter for carrying out large-scale cardiovascular screening. Furthermore, some authors concluded that PCDU test was a predictor of cardiovascular risk with high specificity but low sensitivity, even if in this case a single cut-off for PSV was used [19]. Another point of interest is the choice of the cut-off of PSV as 35 cm/s. In fact, in light of the evidence that the strongest associations with arterial damages were reported using a cut-off of 25 cm/s [21,22,23], our data should not be interpreted as supporting the superiority of this cut-off (35 cm/s) compared to lower thresholds reported in the literature.

Similarly, it was found that the percentage of response to intracavernous PGE1 was negatively correlated with QRISK3 score suggesting that patients with lower erectile response have a higher cardiovascular risk after 10 years. There are no specific studies in this regard, so it could be a first finding of a parameter to be taken into consideration in the evaluation of the patients’ global CV health, to be explored further with further studies. Furthermore, the quantity of drug administered in our cohort of patients during the procedure is not standardized since it is on average approximately 10 µg, with possible adjustments between 2 and 20 µg according to patient characteristics and erectile response.

In the absence of validated questionnaires assessing ED severity, the response to intracavernous PGE1 might provide a rough functional indicator of penile hemodynamic performance. Indeed, previous studies have suggested that the erectile response to pharmacological stimulation is related to erection quality and vascular function, often used for haemodynamic diagnosis of ED and potentially useful combined with IIEF [24,25]. In addition, new scenarios have been recently proposed using shear wave elastography. In detail, Sakamoto et al. [26] evaluated shear wave elastography results of the corpus cavernosum before and after injection in 16 patients undergoing PGE1 testing and reported that the rate of change in shear wave transmission velocity due to PGE1 injection was associated with the degree of erection. Therefore, the rate of change in shear wave transmission velocity in the corpus cavernosum penis could be used as an objective index of erectile phenomenon. However, it is important to emphasize that the PGE1 response cannot be considered a validated surrogate of ED severity and should not replace standardized instruments such as IIEF or EHS. This limitation is particularly relevant in the present study, given the retrospective design and the lack of a fully standardized dosing protocol. Therefore, the interpretation of vascular findings should be made with caution and primarily in terms of hemodynamic assessment rather than clinical severity of ED. Moreover, PGE1 response should be interpreted as a hemodynamic indicator rather than a clinical severity measure.

Regarding classical biochemical risk factors, we found no correlation between LDL and glycaemia, which might reflect the heterogeneous metabolic profiles of the cohort, in the light that levels of LDL and blood glucose are important factors for cardiovascular well-being [27]. Another interesting moderate correlation found was between FSH and cardiovascular risk, also confirmed by the literature, as this hormone influences the development of adipose tissue and the production of inflammatory cytokines, which contribute to the development of atherosclerotic disease [28].

A statistically significant association between pathological PSV and obesity/MetS was not found, contrary to what was expected from studies in the literature [6]. Similarly, even the presence of arterial hypertension was found not to be correlated with pathological PSV value, contrary to what might be expected [29]. Finally, although the presence of hypogonadism was not associated with pathological PSV values, absolute free testosterone levels were significantly lower in the group with pathological PSV. This observation is consistent with the established role of testosterone in modulating sexual function.

To our knowledge, limited data exist dealing with the association between vascular “not-PSV” parameters of PCDU, namely EDV and RI, and cardiovascular risk according to QRISK3 score. As explained above, EDV is a parameter which indicates the amount of flow still present in the artery at the end of the diastolic phase whilst RI expresses the degree of peripheral resistance to flow. In particular, according to our data, we found a moderate positive correlation between EDV and QRISK3 score and a moderate negative correlation between RI and QRISK3 score. In our study, PCDU parameters variations significantly predicted QRISK3 changes, supporting their assessment and report during the PCDU execution. In fact, RI and EDV are parameters of venous alterations, being present both with normal and pathological PSV. In this regard, a patho-physiological role might be imputed to the fibrosis of penis (and corpora cavernosa), which might have a detrimental impact even on venous parameters [30,31].

At multivariate analysis, the PCDU parameters were not independent predictors of cardiovascular risk, likely due to the strong contribution of age, which is a major determinant of the QRISK3 score. The high R^2^ observed in multivariable models is largely driven by age, which represents the main component of the QRISK3 algorithm. In light of these findings, reporting EDV and RI in the clinical report might still be valuable, even if their informative contribution should be interpreted in the context of age and diabetes status, being RI and EDV related—at least partially—with CVD risk.

Moreover, we found that the QRISK3 score was progressively increased according to the number of pathological PCDU parameters. This data might open a new scenario in the whole comprehension of pathology and management of vascular ED, as it might associate arterial and venous parameters in a peculiar anatomical district such as the penis. Therefore, it appears pivotal to perform a complete evaluation of all the vascular parameters of PCDU (namely, PSV, EDV and RI), supporting once again the role of the use of alprostadil in respect to the basal unstimulated evaluation of vascular penile ultrasound. The whole penile vascular function is therefore to be studied, in order to not only perform a correct diagnosis of vascular ED, but also to stratify (or, better, to deeper integrate the stratification) of CVD risk. This might be especially pivotal in patients with normal PSV but pathological venous parameters and, on the other hand, in patients with pathological PSV in whom venous parameters are often omitted in the PCDU report.

A potential methodological concern relates to the use of QRISK3 as an outcome measure. Since QRISK3 incorporates variables such as age, blood pressure, lipid profile, and diabetes mellitus, models including these variables will naturally demonstrate high discriminative performance. Therefore, the present analyses should not be interpreted as attempts to construct a predictive model for QRISK3 itself. Rather, our aim was to explore whether PCDU parameters were associated with estimated cardiovascular risk categories derived from an established risk algorithm.

Further studies are needed in order to clarify and confirm this evidence, in particular evaluating other ultrasound parameters possibly associated with CVD risk (such as the cavernous intima-media thickness and the systolic rise time of cavernous artery) and the associations with other metabolic, cardiovascular and hormonal parameters, as well as the association with the response to therapy for ED. Moreover, in future studies it might be useful to evaluate the association between PCDU parameters and coronary CT scan [32] (especially in such patients without PCVE, for whom the coronary CT scan represents the gold standard [12]). Finally, future studies might evaluate the reliability of age-adjusted PSV, as previously reported [33,34], as well as the evaluation of other cardiovascular and cardiometabolic parameters (recently evaluated in hypogonadal diabetic patients) [35,36].

Our findings do not support the use of PSV as a standalone cardiovascular screening tool, but rather as a physiological marker of systemic vascular impairment.

The limitations of our study include: the retrospective nature of the study, which does not allow definitive conclusions to be drawn on the causal relationship between the observed associations; the monocentric nature of the study; the lack of standardized etiological classification (such as reported by Pozzi et al. [37])—although patients referred for PCDU are typically those with suspected vascular ED, the possibility of mixed or non-vascular components cannot be completely excluded; the lack of data addressing marital history, relationship status, education, socioeconomic status, and sexual orientation. Furthermore, although the examinations were performed by two experienced operators following a standardized protocol, PCDU remains an operator-dependent technique and inter-observer variability could not be formally assessed in this retrospective study. In addition, another limitation of this study is the lack of standardized questionnaires assessing erectile dysfunction severity, such as IIEF-5, EHS, or other validated instruments, and this might limit the possibility to directly correlate vascular findings with clinical symptom burden. Because of the retrospective design and the long observation period, these tools were not systematically recorded for all patients. Therefore, the present analysis focused primarily on vascular parameters obtained from PCDU rather than on the clinical grading of ED severity. Finally, because the study was conducted in a tertiary referral andrology center, the patient population might not fully represent the general population of men with ED evaluated in primary care settings (the tertiary referral nature of our center might in fact have introduced selection bias, as patients referred for PCDU often represent a clinically complex population with suspected vascular ED). Therefore, caution should be used when generalizing these findings and interpreting the external validity of such results.

## 5. Conclusions

PCDU represents a valuable tool for the vascular evaluation of patients with ED. In this study, we reported a correlation of both arterial and venous parameters of PCDU with the CVD risk of patients with ED. This data opens a new scenario in the setting of the PCDU, underlying the importance of a complete evaluation of vascular parameters during PCDU, especially in patients without PCVE. In particular, reports of PCDU should be interpreted primarily as markers of systemic vascular dysfunction in selected patients with ED, rather than a general cardiovascular screening strategy. Nevertheless, PCDU does not represent a fully validated substitute for validated questionnaires.

## Figures and Tables

**Table 1:** General data regarding the patients enrolled and the differences in such parameters according to the PSV value. Numbers of patients with the reported variable were different due to missing data handling. Abbreviations: cFT: calculated free testosterone; FSH: follicle-stimulating hormone; HDL: high density lipoproteins; LH: luteinizing hormone; N: number of patients with available data; PCVE: previous cardiovascular events; PSA: prostate specific antigen; SHBG: sex hormone binding globulin; TGL: triglycerides; TSH: thyroid-stimulating hormone; TT: total testosterone.

Variable	N	Overall	PSV < 35 cm/s	PSV ≥ 35 cm/s	*p*-Value
Age, years, median [IQR]	275	57 [48–65]	62 [54–67]	55 [48–64]	**0.001**
QRISK^®^ 10 y, %, median [IQR]	253	10.7 [5.8–16.6]	13.6 [10.1–14.6]	10.0 [5.5–15.5]	**0.006**
Mean PSV, cm/s, median [IQR]	275	49 [37–61]	27.5 [23.0–31.5]	55 [45–64]	**<0.001**
Mean EDV, cm/s, median [IQR]	275	3.0 [0.0–6.5]	4.0 [0.0–6.5]	2.5 [−2.9–6.5]	0.115
Mean RI, median [IQR]	270	0.91 [0.83–1.00]	0.82 [0.77–0.95]	0.95 [0.86–1.00]	**<0.001**
Response to PGE1, %, median [IQR]	275	80 [70–100]	65 [50–80]	83 [70–100]	**<0.001**
PCVE %	274	6.2	13.6	4.2	**0.008**
Diabetes mellitus, %	275	9.5	16.9	7.4	**0.026**
Hypertension, %	275	25.8	22.0	26.9	0.454
Dyslipidaemia, %	275	18.2	22.0	17.1	0.387
Obesity, %	275	11.6	13.6	11.1	0.603
Hypogonadism, %	275	10.5	6.8	11.6	0.288
Hypothyroidism, %	275	5.1	1.7	6.0	0.181
Metabolic syndrome, %	275	8.0	6.8	8.3	0.697
TT, nmol/L, median [IQR]	137	13.53 [8.93–16.98]	12.84 [8.16–16.15]	13.5 [9.3–17.3]	0.479
SHBG, nmol/L, median [IQR]	105	36.7 [27.3–52.7]	41.8 [29.5–55.9]	36.7 [27.1–52.5]	0.603
cFT, nmol/L, median [IQR]	102	0.23 [0.18–0.30]	0.20 [0.13–0.26]	0.24 [0.19–0.31]	**0.040**
TSH, IU/L, median [IQR]	115	1.71 [1.14–2.48]	1.50 [1.01–2.35]	1.73 [1.17–2.49]	0.311
LH, IU/L, median [IQR]	131	4.7 [3.4–7.5]	4.0 [3.0–6.1]	5.0 [3.6–7.6]	0.133
FSH, IU/L, median [IQR]	83	6.1 [4.3–12.0]	5.3 [4.3–6.1]	6.3 [4.3–12.5]	0.507
PSA, μg/L, median [IQR]	100	1.050 [0.502–1.995]	0.990 [0.350–1.391]	1.110 [0.540–2.070]	0.231
TGL, mg/dl, median [IQR]	117	102 [79–133]	99 [83–135]	102 [79–133]	0.637
Total cholest, mg/dl, median [IQR]	122	199 [171–227]	204 [165–229]	199 [174–227]	0.943
HDL, mg/dl, median [IQR]	118	50 [43–56]	50 [43–52]	50 [43–56]	0.789
LDL, mg/dl, median [IQR]	116	131 [100–153]	135 [97–155]	130 [100–153]	0.709
BMI, kg/m^2^, median [IQR]	78	28.7 [25.4–32.4]	28.6 [26.2–34-9]	28.7 [25.2–31.6]	0.560
Glycaemia, mg/dl, median [IQR]	119	97 [89–108]	98 [91–109]	97 [88–107]	0.561

**Table 2:** Correlations between QRISK 10, mean PSV and response to PGE1 and main clinical and laboratory parameters. Abbreviations: BMI: body mass index; cFT: calculated free testosterone; FSH: follicle-stimulating hormone; HDL: high density lipoproteins; LDL: low density lipoproteins; LH: luteinizing hormone; N: number of patients with available data; PCVE: previous cardiovascular events; PSA: prostate specific antigen; PGE1: alprostadil; PSV: peak of systolic velocity; SHBG: sex hormone binding globulin; TG: triglycerides; TSH: thyroid-stimulating hormone; TT: total testosterone. * *p* < 0.05, ** *p* < 0.01.

Variable	QRISK 10	Mean PSV	Response to PGE1
Age	0.902 **	−0.217 **	−0.361 **
QRISK 10	-	−0.203 **	−0.272 **
Mean PSV	−0.203 **	-	0.441 **
Response to PGE1	−0.272 **	0.441 **	-
Mean EDV	0.175 **	−0.173 **	−0.633 **
Mean RI	−0.191 **	0.357 **	0.674 **
TT	−0.038	−0.093	−0.071
SHBG	0.262 **	−0.046	−0.121
cFT	−0.133	−0.043	0.153
TSH	−0.116	−0.006	0.094
LH	0.149	0.034	−0.082
FSH	0.281 *	0.028	−0.158
TG	0.094	−0.079	0.045
Total cholesterol	0.175	−0.071	0.030
HDL	−0.053	0.043	−0.101
LDL	0.211 *	−0.131	−0.032
BMI	0.119	−0.209	−0.041
Glycemia	0.280 **	−0.159	−0.093

## Data Availability

The data that support the findings of this study are available from the corresponding author upon reasonable request.

## Supplementary Materials

The following supporting information can be downloaded at: https://www.mdpi.com/article/10.3390/jcm15072722/s1, Table S1: Linear regression analyses evaluating the association between penile Doppler parameters and estimated cardiovascular risk (QRISK3). Univariate and multivariable models are presented. Age and diabetes mellitus were included in multivariable models as major clinical confounders. R^2^ values represent the proportion of variance in QRISK3 explained by the regression model; Figure S1.
