# Supplementary material for: Association Between Parameters of Penile Doppler Ultrasound and Cardiovascular Risk in Patients with Erectile Dysfunction: A Single-Center Retrospective Study
Source: J Clin Med. 2026 Apr 3;15(7):2722. doi: 10.3390/jcm15072722 (PMC13073985; doi:10.3390/jcm15072722)
Supplement: Supplementary file 1 [file jcm-15-02722-s001.zip › jcm-4211159-supplementary.pdf]

Supplementary Table S1. Linear regression analyses evaluating the association between penile Doppler parameters and estimated cardiovascular risk (QRISK3). Univariate and multivariable models are presented. Age and diabetes mellitus were included in multivariable models as major clinical confounders. R^2 values represent the proportion of variance in QRISK3 explained by the regression model.

Variable	R^2	β	B	95% CI	p-value
Univariate	0.04				
PSV, cm/s		-0.200	-0.090	-0.144; -0.035	0.001
Multivariate	0.764				
PSV, cm/s		-0.033	-0.015	-0.046; 0.017	0.360
Age, year		0.854	0.576	0.532; 0.620	<0.001
Diabetes mellitus		0.177	5.047	3.295; 6.799	<0.001
Response to PGE1, %		0.045	0.017	-0.010; 0.045	0.220
Univariate	0.024				
EDV, cm/s	.	0.155	0.174	0.036; 0.320	0.014
Multivariate	0.763				
EDV, cm/s		0.013	0.014	-0.067; 0.096	0.731
Age, year		0.855	0.577	0.533; 0.620	<0.001
Diabetes mellitus		0.181	5.168	3.424; 6.911	<0.001
Response to PGE1, %		0.037	0.014	-0.01; 0.043	0.337
Univariate	0.032				
RI, absolute		-0.180	-13.542	-22.85; -4.229	0.005
Multivariate	0.760				
RI, absolute		0.011	0.836	-5.111; 6.783	0.782
Age, year		0.859	0.577	0.533; 0.621	<0.001
Diabetes mellitus		0.169	5.016	3.177; 6.855	<0.001
Response to PGE1, %		0.020	0.008	-0.025, 0.041	0.621
