# Supplementary figures and images for: Association Between Parameters of Penile Doppler Ultrasound and Cardiovascular Risk in Patients with Erectile Dysfunction: A Single-Center Retrospective Study
Source: J Clin Med. 2026 Apr 3;15(7):2722. doi: 10.3390/jcm15072722 (PMC13073985; doi:10.3390/jcm15072722)

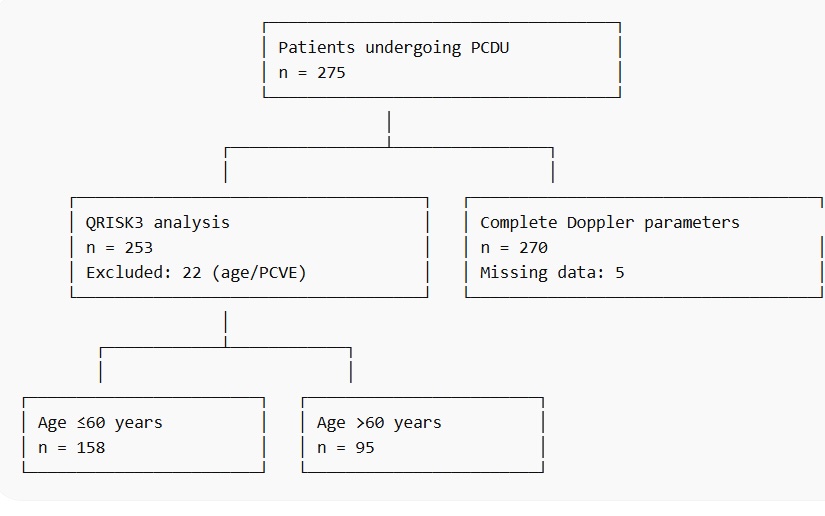

Supplement: Supplementary file 1 [file jcm-15-02722-s001.zip › jcm-4211159-Figure S1.jpg]
